# Influence of Electronic Non-Equilibrium on Energy Distribution and Dissipation in Aluminum Studied with an Extended Two-Temperature Model

**DOI:** 10.3390/nano12101655

**Published:** 2022-05-12

**Authors:** Markus Uehlein, Sebastian T. Weber, Baerbel Rethfeld

**Affiliations:** Department of Physics and Research Center OPTIMAS, Technische Universität Kaiserslautern, Erwin-Schrödinger-Straße 46, 67663 Kaiserslautern, Germany; weber@physik.uni-kl.de (S.T.W.); rethfeld@physik.uni-kl.de (B.R.)

**Keywords:** ultrafast dynamics, temperature-based model, electronic non-equilibrium, high-energetic electrons, femtosecond laser pulse, aluminum

## Abstract

When an ultrashort laser pulse excites a metal surface, only a few of all the free electrons absorb a photon. The resulting non-equilibrium electron energy distribution thermalizes quickly to a hot Fermi distribution. The further energy dissipation is usually described in the framework of a two-temperature model, considering the phonons of the crystal lattice as a second subsystem. Here, we present an extension of the two-temperature model including the non-equilibrium electrons as a third subsystem. The model was proposed initially by E. Carpene and later improved by G.D. Tsibidis. We introduce further refinements, in particular, a temperature-dependent electron–electron thermalization time and an extended energy interval for the excitation function. We show results comparing the transient energy densities as well as the energy-transfer rates of the original equilibrium two-temperature description and the improved extended two-temperature model, respectively. Looking at the energy distribution of all electrons, we find good agreement in the non-equilibrium distribution of the extended two-temperature model with results from a kinetic description solving full Boltzmann collision integrals. The model provides a convenient tool to trace non-equilibrium electrons at small computational effort. As an example, we determine the dynamics of high-energy electrons observable in photo-electron spectroscopy. The comparison of the calculated spectral densities with experimental results demonstrates the necessity of considering electronic non-equilibrium distributions and electron–electron thermalization processes in time- and energy-resolved analyses.

## 1. Introduction

The relevance of lasers in current research is immense. In particular, ultrashort pulses in the femtosecond regime are of enormous importance for the processing of various materials, from metals [1,2,3], semiconductors and insulators [4,5] to biological tissues [6,7], and for technical [8,9,10], chemical [11] or medical [12] applications. The response of the solid matter to ultrafast excitation is also of great interest from the fundamental point of view. In particular, the time range of a few tens of femtoseconds after laser excitation is of importance, since intrinsic collision processes within the material take place on these timescales [13,14,15]. Experimentally, time-resolved measurements give access to fundamental interaction processes within solid matter [13,16,17,18,19,20,21,22,23,24]. On the theoretical side, non-equilibrium electron kinetics can be traced with various methods, e.g., Monte-Carlo simulations [25,26], time-dependent density functional theory simulations [27,28], kinetic equations [29,30,31], or Boltzmann collision integrals [17,21,32,33,34,35,36]. Such models are capable of describing the pathway of thermalization from the initial laser-induced non-equilibrium electron distribution to a Fermi distribution of a well-defined temperature. Since they are, however, rather complex and numerically expensive, their costs and benefits may not be well-balanced, when the details of the energy distribution can be neglected. An approximate consideration of the laser-induced non-equilibrium electronic energy distribution can be sufficient in many cases, when qualitative aspects of the non-equilibrium are studied, or an integration over distinct energy ranges is possible.

The non-equilibrium between electrons and phonons is approximately captured by the two-temperature model (TTM) first established by Anisimov et al. [37]. It is well-known, well-validated on time scales of thermalized electrons [2,38], and easily extendable [39,40,41,42]. Several modifications of the TTM have been proposed to capture the main features of the electronic or phononic non-equilibrium [17,43,44,45,46]. Here, we study a simplified description of electronic non-equilibrium. We investigate the so-called extended two-temperature model (eTTM), which is based on the original proposal of Carpene [44], which was later extended by Tsibidis [43]. We further improve the model and study the resulting energy-distribution and energy-dissipation processes.

Describing non-equilibrium effects with help of the eTTM has a strong benefit over full kinetic calculations in terms of numerical costs and duration. It runs in a few minutes on one core of a desktop computer, whereas typical simulations using Boltzmann collision integrals require several hours on a high-performance cluster.

In this paper, we first recall the basic idea of the eTTM, describe the temporal development of the non-equilibrium subsystem, and introduce the changes made in comparison to [43]. Then, we show selected results of the eTTM and compare to the corresponding results of the TTM. Next, we investigate the influence of the presented improvements by showing details of the calculated non-equilibrium distribution. Finally, we compare the results of photon-absorption using the TTM, eTTM and a Boltzmann calculation. The importance of the description of the non-equilibrium becomes apparent when the dynamics of the electron number density in a specific energy range above the Fermi edge is evaluated. This quantity is accessible by time-resolved two-photon photoemission measurements.

## 2. Theoretical Model

### 2.1. Two Temperatures and a Non-Equilibrium System

When a laser irradiates a metal, photons are absorbed by the electrons in the solid, causing an increase of the electronic temperature. Through subsequent electron–phonon collisions, energy is transferred to the lattice, leading to a joint temperature of electrons and phonons. Usually, this is described by the well-known two-temperature model (TTM) [37,47]. It consists of two differential equations for the change in the internal energy density *u* of the electrons and the phonons, respectively, with the time *t*

(1a) $$\frac{du_{\mathrm{el}}}{dt}=c_{\mathrm{el}}\frac{\partial T_{\mathrm{el}}}{\partial t}=-g\left(T_{\mathrm{el}}-T_{\mathrm{ph}}\right)+s(t),$$

(1b) $$\frac{du_{\mathrm{ph}}}{dt}=c_{\mathrm{ph}}\frac{\partial T_{\mathrm{ph}}}{\partial t}=g\left(T_{\mathrm{el}}-T_{\mathrm{ph}}\right),$$

where the index “el” stands for the electrons and “ph” for the phonons. The change in the internal energy *u* can be reformulated to a change in the temperatures *T*, using heat capacities *c*. For more information about heat capacity, see Appendix A.1.

The laser excites only the electronic system and is represented through the source term s(t), describing the laser power density. More details regarding the laser are listed in Appendix A.2. The energy transfer between electrons and phonons enters both equations. It consists of the temperature difference between the subsystems and a proportionality factor *g*, called electron–phonon coupling parameter.

Zooming to ultrashort timescales, the absorption of the photons is, however, more complex [16,48,49]. When the energy distribution of electrons becomes relevant, the energy density alone is not sufficient to describe the excitation of the system. In fact, only a small fraction of all electrons can absorb photons. At moderate intensities, only single photons are absorbed. The absorbing electrons are located in a range of one photon energy below the Fermi edge and will, after excitation, populate states in a range of one photon energy above the Fermi edge [48,49]. Due to Pauli blocking, other transitions are unlikely to occur at room temperature. As a result, the Fermi distribution is strongly disturbed and no temperature can be defined for the electrons. The excited non-equilibrium electrons thermalize back to equilibrium, mainly by collisions with other electrons.

**Figure 1:**
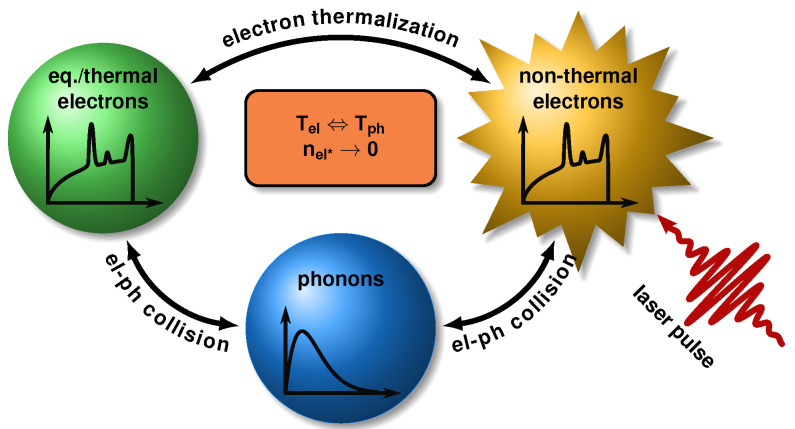
Schematic illustration of the subsystems and the interactions in the extended two-temperature model. The laser generates non-equilibrium (non-thermal) electrons. They thermalize by collisions with the unaffected (thermal) electrons. Thus, the particle density of excited electrons $n_{\mathrm{el}*}$ decreases during thermalization. Moreover, collisions of electrons with phonons lead to a heating of the crystal lattice. Both non-thermal and thermal electrons contribute to this relaxation process, which results in a joint electron and phonon temperature.

This initial non-equilibrium dynamics cannot be traced by the TTM. Thus, Carpene [44], and later Tsibidis [43], developed the extended two-temperature model (eTTM) depicted in Figure 1. Like the TTM, it traces the temperature of the electrons and phonons (symbolized with the blue and green balls). However, the laser does not increase the temperature of the equilibrium electrons directly, but excites high-energy non-equilibrium electrons above and associated holes below the Fermi edge. They are treated as a third system and are marked with a star-like shape. All systems can interact with each other. These interactions are shown as arrows.

The model was introduced first by Carpene, with a constant density of states (DOS) around the Fermi edge [44]. This allows to solve large parts of the model analytically. Tsibidis improved this model by considering a more realistic DOS [43]. Consequently, some components of the model can only be solved numerically. We applied the model of Tsibidis in order to compare with full kinetic calculations. Therefore, we introduced slight improvements, which will be discussed in Section 2.3.

The extended two-temperature model, like the conventional TTM, consists of a differential equation for the internal energy density *u* of the thermal electrons and one for the internal energy density of phonons, both including the interaction with the non-thermal electrons,

(2a) $$\frac{du_{\mathrm{el}}}{dt}=-g\left(T_{\mathrm{el}}-T_{\mathrm{ph}}\right)+\frac{\partial u_{\mathrm{el}*-\mathrm{el}}}{\partial t}=:-\Pi_{\mathrm{el}-\mathrm{ph}}\left(t\right)+\Xi_{\mathrm{el}*-\mathrm{el}}\left(t\right),$$

(2b) $$\frac{du_{\mathrm{ph}}}{dt}=g\left(T_{\mathrm{el}}-T_{\mathrm{ph}}\right)+\frac{\partial u_{\mathrm{el}*-\mathrm{ph}}}{\partial t}=:\Pi_{\mathrm{el}-\mathrm{ph}}\left(t\right)+\Xi_{\mathrm{el}*-\mathrm{ph}}\left(t\right),$$

where the index “el” denotes the thermal electrons as before, and “el${}^{*}$” the non-thermal electron system. Each of the differential equations contains the electron–phonon coupling term, abbreviated here as $\Pi_{\mathrm{el}-\mathrm{ph}}$. The term describes the coupling between the thermalized electrons and the phonons, like in the TTM, cf. the first terms on the right-hand side of Equations (1a) and (1b). In the eTTM, the excitation term s(t) is replaced by a coupling term of the non-thermal electrons to the thermalized electrons and phonons, called $\Xi_{\mathrm{el}*-\mathrm{el}}=\frac{\partial u_{\mathrm{el}*-\mathrm{el}}}{\partial t}$ and $\Xi_{\mathrm{el}*-\mathrm{ph}}=\frac{\partial u_{\mathrm{el}*-\mathrm{ph}}}{\partial t}$, respectively. The Ξ-terms and the description and dynamics of the non-equilibrium system are explained in detail in the next section.

### 2.2. Time Evolution of the Non-Equilibrium System

In order to describe the theory of this model as closely as possible to its numerical implementation, time is considered as a discrete quantity. Here, Δt is the time step of this discretization. When photons with energy $E_{\mathrm{pt}}$ reach the material within the time interval $\left[t^{\prime },t^{\prime }+\Delta t\right]$, they are absorbed and non-equilibrium electrons are generated. These non-equilibrium electrons and the associated holes are described by the difference from the current Fermi distribution, given as $f_{\mathrm{Fermi}}\left(E\right):=f_{\mathrm{Fermi}}\left(t^{\prime },E\right):=f_{\mathrm{Fermi}}\left(T_{\mathrm{el}}\left(t^{\prime }\right),E\right)$, which describes the thermalized electronic background. It is assumed that this difference to equilibrium, the so-called excitation function, can be described by

(3) $$\Delta f_{L}\left(t^{\prime },E\right)=\delta \left(t^{\prime }\right)\left\{f_{\mathrm{Fermi}}\left(E-E_{\mathrm{pt}}\right)\left[1-f_{\mathrm{Fermi}}\left(E\right)\right]-f_{\mathrm{Fermi}}\left(E\right)\left[1-f_{\mathrm{Fermi}}\left(E+E_{\mathrm{pt}}\right)\right]\right\}.$$

This excitation function, driven by the laser, generates non-equilibrium electrons within the time-interval $\left[t^{\prime },t^{\prime }+\Delta t\right]$ [43]. It modulates a step-like structure around the Fermi edge, which builds up when the absorption under the consideration of the Pauli principle takes place. This behavior is known also from kinetic simulations [33]. For every observed energy level *E*, two possible processes can happen. Electrons from the energetically lower level $E-E_{\mathrm{pt}}$ can absorb one photon, leading to an increase in the distribution at energy *E*. On the other hand, already-existing electrons at energy *E* can scatter into the higher level $E+E_{\mathrm{pt}}$. The probability of the first process is proportional to the term $f_{\mathrm{Fermi}}\left(E-E_{\mathrm{pt}}\right)\left[1-f_{\mathrm{Fermi}}\left(E\right)\right]$, while the probability of the latter process can be expressed through $f_{\mathrm{Fermi}}\left(E\right)\left[1-f_{\mathrm{Fermi}}\left(E+E_{\mathrm{pt}}\right)\right]$. The generated steps have a width that corresponds to the photon energy $E_{\mathrm{pt}}$. The amplitude of the step δ is given by the comparison

(4) $$\int_{-\infty }^{\infty }\Delta f_{L}\left(t^{\prime },E\right)\;D\left(E\right)\;E\;dE=s\left(t^{\prime }\right)\Delta t$$

with the laser power density $s(t^{\prime })$ of the TTM. The energy-dependent D(E) denotes the density of states of the electrons. In this work, the energy zero is set to the Fermi edge, so the integration starts at negative energy.

At time $t>t^{\prime }$, the electrons generated in the time interval $\left[t^{\prime },t^{\prime }+\Delta t\right]$ thermalize by collisions with electrons and phonons. According to [43,44], the dynamics of the excitation function within each of those time intervals can be described by

(5) $$\Delta f^{\prime }\left(t,t^{\prime },E\right)=\Delta f_{L}\left(t^{\prime },E\right)\exp\left(-\frac{t-t^{\prime }}{\tau_{\mathrm{el}*-\mathrm{el}}}-\frac{t-t^{\prime }}{\tau_{\mathrm{el}*-\mathrm{ph}}}\right)\;,$$

using energy relaxation times $\tau_{\mathrm{el}*-\mathrm{el}}$ and $\;\tau_{\mathrm{el}*-\mathrm{ph}}$. We use an energy- and temperature-dependent electron–electron relaxation time

(6) $$\tau_{\mathrm{el}*-\mathrm{el}}\left(E,T\right)=\tau_{0}\frac{E_{F}^{2}}{{\left(E-E_{F}\right)}^{2}+{\left(\pi k_{B}T_{\mathrm{el}}\right)}^{2}}\;,$$

with pre-factor $\tau_{0}$ approximating the lifetime obtained from Fermi-liquid theory [33,50,51]. In contrast to the relaxation time used in [43], which includes only a dependence on energy, Equation (6) contains also a temperature-dependent term. It accounts for the broadening of the Fermi edge at elevated temperatures and prevents singularities of the relaxation time at the Fermi edge. The electron–phonon relaxation time $\tau_{\mathrm{el}*-\mathrm{ph}}$ is calculated according to [19,44]. More details about the parameters applied are given in Appendix A.3.

For various investigations, it is useful to calculate the distribution present at a certain time *t*, which describes all electrons, thermalized and non-thermalized together, e.g., it enters the total electron number in a specific energy range, which is also observable in experimental photoemission results, see Section 3.4. The total electron distribution at time *t*

(7) $$f^{\mathrm{tot}}\left(t,E\right)=f_{\mathrm{Fermi}}\left(t,E\right)+\Delta f\left(t,E\right)=f_{\mathrm{Fermi}}\left(t,E\right)+\sum_{\begin{array}{c} t^{\prime }=0,\\ t^{\prime }+=\Delta t \end{array}}^{t}\Delta f^{\prime }\left(t,t^{\prime },E\right)$$

is given by adding the distributions of the thermalized and the non-thermalized electrons. The thermalized electrons are described by a time-dependent Fermi distribution, $f_{\mathrm{Fermi}}\left(t,E\right)$. The distribution of the non-thermalized electrons, Δf(t,E), is obtained by the sum of all thermalizing excitation functions $\Delta f^{\prime }\left(t,t^{\prime },E\right)$, see Equation (5), for all previous time intervals in steps of Δt.

With help of the time-dependent excitation function Equation (5), the total energy transfer from the system of the non-equilibrium electrons to the equilibrium systems can be calculated. At time *t*, the non-equilibrium electrons generated in the time interval $\left[t^{\prime },t^{\prime }+\Delta t\right]$ provide the contribution

(8) $$\begin{array}{cc} \frac{\partial u^{\prime }\left(t,t^{\prime }\right)}{\partial t} & =-\frac{\partial }{\partial t}\int_{-\infty }^{\infty }\Delta f^{\prime }\left(t,t^{\prime },E\right)\;D\left(E\right)\;E\;dE\\ \; & =-\int_{-\infty }^{\infty }\left(-\frac{1}{\tau_{\mathrm{el}*-\mathrm{el}}}-\frac{1}{\tau_{\mathrm{el}*-\mathrm{ph}}}\right)\Delta f_{L}\left(t^{\prime },E\right)\exp\left(-\frac{t-t^{\prime }}{\tau_{\mathrm{el}*-\mathrm{el}}}-\frac{t-t^{\prime }}{\tau_{\mathrm{el}*-\mathrm{ph}}}\right)\;D\left(E\right)\;E\;dE\\ \; & =:\frac{\partial u_{\mathrm{el}*-\mathrm{el}}^{\prime }\left(t,t^{\prime }\right)}{\partial t}+\frac{\partial u_{\mathrm{el}*-\mathrm{ph}}^{\prime }\left(t,t^{\prime }\right)}{\partial t}=:\Xi_{\mathrm{el}*-\mathrm{el}}^{\prime }\left(t,t^{\prime }\right)+\Xi_{\mathrm{el}*-\mathrm{ph}}^{\prime }\left(t,t^{\prime }\right). \end{array}$$

It can be split up into one contribution entering the equilibrium electrons $\Xi_{\mathrm{el}*-\mathrm{el}}^{\prime }\left(t,t^{\prime }\right)$ and one contribution entering the phonons $\Xi_{\mathrm{el}*-\mathrm{ph}}^{\prime }\left(t,t^{\prime }\right)$. The total energy transfer rates from non-equilibrium electrons to the respective equilibrium subsystems entering (2) are given by the sum over all previous time steps,

(9) $$\Xi_{\mathrm{el}}{}^{*}-\mathrm{el}/\mathrm{ph}\left(t\right):=\frac{\partial u_{\mathrm{el}}{}^{*}-\mathrm{el}/\mathrm{ph}\left(t\right)}{\partial t}=\sum_{\begin{array}{c} t^{\prime }=0,\\ t^{\prime }+=\Delta t \end{array}}^{t}\Xi_{\mathrm{el}}{}^{*}{-\mathrm{el}/\mathrm{ph}}^{\prime }\left(t,t^{\prime }\right)=\sum_{\begin{array}{c} t^{\prime }=0,\\ t^{\prime }+=\Delta t \end{array}}^{t}\frac{\partial u_{\mathrm{el}}{}^{*}{-\mathrm{el}/\mathrm{ph}}^{\prime }\left(t,t^{\prime }\right)}{\partial t}.$$

### 2.3. Improvements as Compared to the Work of G.D. Tsibidis

The model presented in the previous section is a slightly improved version of the eTTM published in [43]. Besides the enhanced electron–electron relaxation time (see Equation (6)), the integration limits are also extended. In contrast to the eTTMs published so far [43,44], the entire energy range is considered here for the integrations in Equations (4) and (8). The previous models only integrated over the range $\left[E_{F}-E_{\mathrm{pt}},\;E_{F}+E_{\mathrm{pt}}\right]$ around the Fermi edge $E_{F}$, which entails an non-physical limitation of absorption at temperatures above absolute zero. The influence of these improvements on the distribution function of the electrons are discussed in Section 3.2.

## 3. Results

The model is applied to aluminum as an example material. The material parameters and the parameters of the Gaussian laser pulse are listed in Appendix A, unless otherwise specified. In the following, all time-dependent figures show the temporal shape of the laser pulse in grey.

The description given here is valid for a homogeneously heated, thin metal film, because any transport through the material has been neglected. Tsibidis considered the transport of thermal electrons; however, he neglected the heat transport of non-equilibrium electrons [43]. Though its influence seems to be rather relevant [24,52,53], there is, to the best of our knowledge, no description capturing non-equilibrium transport in the framework of a temperature-based model.

### 3.1. Temperature and Energies

In the following, we investigate the temperatures and energies of the subsystems, as well as the energy transfer between them. Comparing results of the eTTM and TTM, we highlight the influences of non-equilibrium electrons.

**Figure 2:**
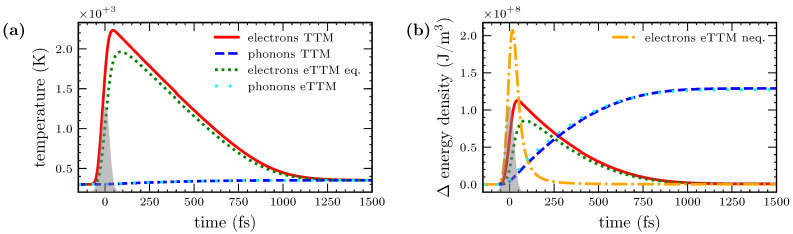
(**a**) Temperatures of electrons and phonons. The results of a TTM simulation are compared to the results of an eTTM simulation. The shape of the laser pulse is sketched in gray. It is visible that the maximum temperature of the electrons in the TTM simulation is higher than the maximum electron temperature obtained with the eTTM simulation. At this stage, part of the energy is kept in the non-thermal electrons of the eTTM. (**b**) Energy density of all subsystems of TTM and eTTM in dependence on time, plotted as difference to the initial values. Due to the connection between energy density and temperature, the dynamics resemble the temperature dynamics. Additionally, the energy density of the non-equilibrium electrons in the eTTM is shown in the yellow dashed-dotted line. It is clearly visible that non-equilibrium electrons are generated directly by the laser and thermalize in the next hundreds of femtoseconds.

Figure 2a compares the dynamics of the temperatures of electrons and phonons obtained with both models for the same absorbed fluence. Please notice that no temperature can be assigned to the non-equilibrium electrons. Therefore, only the temperature of the equilibrium background is shown for the eTTM. The electron temperatures of both models show the initial rise through the irradiating laser pulse and the relaxation to a joint temperature of electrons and phonons. As expected and desired, there is no significant difference between the temperatures of the TTM and eTTM simulations. However, the maximum temperature of the eTTM is lower than for the TTM and it takes longer to reach this maximum. This is caused by the indirect heating of the equilibrium electrons by the laser through the non-equilibrium system. In Figure 2a, no differences in the temperature rise of the phonons are observed.

In contrast to the temperature, the energy density of the non-equilibrium electrons is well-defined. This allows us to compare the dynamics of the non-equilibrium and the equilibrium systems. Figure 2b depicts the energy densities of all the subsystems of both models in dependence on time. They are plotted as a difference to their initial value, before the laser pulse. The change in the energy densities of the equilibrium electrons and phonons can be directly connected to their respective temperatures via their heat capacity. Therefore, the energy dynamics of those systems are qualitatively equal to the dynamics shown before, cf. Figure 2a. The heat capacity of the phonons is much higher than that of the electrons, leading to a much higher energy gain for the phonons than for the electrons at the same final temperature. The contribution of the non-equilibrium, which could not been shown in Figure 2a, is clearly visible in Figure 2b. It rises directly during irradiation, and decays within less than 200 fs delayed to the laser pulse. This corresponds to thermalization times extracted from two-photon photoemission experiments [24]. The peak energy contained in the non-equilibrium electrons is more than two times larger than the peak energy in the equilibrium electron system. This is a consequence of the laser heating being faster than the energy loss to the equilibrium electrons.

**Figure 3:**
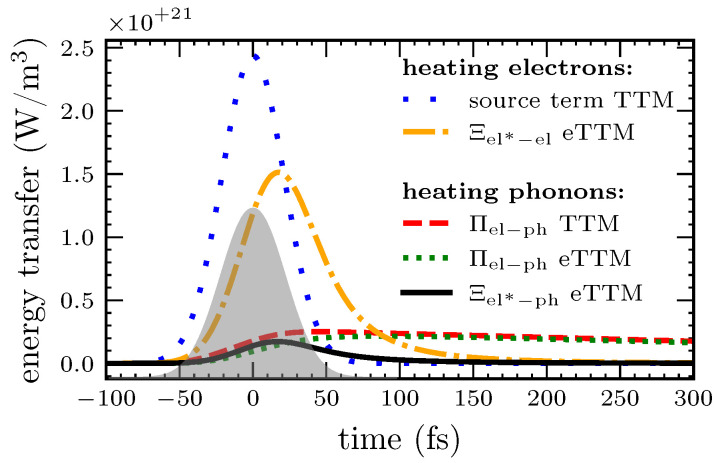
Dynamics of the rate of energy transfer in dependence of time, comparing a TTM and eTTM simulation. The notation corresponds to the definitions introduced in Equation (2). The source term of the TTM, i.e., the laser power density s(t), is shown in the blue loosely dotted line. In the eTTM, the increase in electronic temperature is mediated by interaction with non-equilibrium electrons, described by $\Xi_{\mathrm{el}*-\mathrm{el}}$ and shown with a yellow dashed-dotted line. It lasts considerably longer than the direct laser heating of the TTM. The heating of the phonons is described in the TTM with the electron–phonon coupling term, here denoted as $\Pi_{\mathrm{el}-\mathrm{ph}}$ and depicted with a red dashed line. It is rather similar to the energy transfer from equilibrium electrons to phonons in the eTTM, described by the same term, $\Pi_{\mathrm{el}-\mathrm{ph}}$, and depicted with a green dotted line. Only the initial heating of phonons by equilibrium electrons is slightly reduced. This is due to the lower temperature of electrons in the eTTM; compare Figure 2a. However, the non-equilibrium electrons provide an additional heating mechanism of the phonons in the eTTM, denoted as $\Xi_{\mathrm{el}*-\mathrm{ph}}$ and depicted with a black solid line.

To investigate the coupling strengths between the subsystems, Figure 3 shows the energy transfer rates between them in dependence on time. The distinct coupling terms describe the energy density per time transferred from one subsystem to the other; compare Equations (1a) and (2) and Figure 1. Generally, the energy transfer rates to the electrons are higher than those to the phonons, i.e., the electron systems are heated faster than they lose energy to the phonons. In the case of the TTM, the heating of the equilibrium electrons occurs through the laser source term and follows, thus, exactly the temporal shape of the laser pulse. In the case of the eTTM, the laser heats the non-equilibrium electrons (not shown), which subsequently transfer their energy to the equilibrium electrons and to the phonons, described through the terms $\Xi_{\mathrm{el}*-\mathrm{el}}$ and $\Xi_{\mathrm{el}*-\mathrm{ph}}$, respectively, which are both shown in Figure 3. They are slightly delayed as compared to the laser pulse; however, their integrals sum up to the same total energy density as delivered from the laser source in the case of the TTM. The coupling of the equilibrium electrons to the phonons, $\Pi_{\mathrm{el}-\mathrm{ph}}$, is nearly identical in the TTM and the eTTM, respectively.

### 3.2. Electron Distribution Function

**Figure 4:**
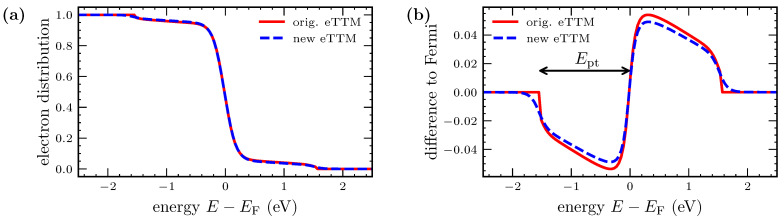
Electron distributions at the maximum of the laser pulse. The original eTTM by Tsibidis [43] is compared to the new and modified version presented in this paper. Figure (**a**) shows the electron distribution, Figure (**b**) the difference to the equilibrium background. The step is visible and has a width that equals the photon energy $E_{\mathrm{pt}}=1.55\;e\;V$, as was explained in the theory part of the model (see Section 2). While the differences in total are small (see (**a**)), the new model prevents the unphysical kink at the edges of the laser-disturbed energy range by distributing the energy of the laser over a wider energy range (see (**b**)).

An important function is the electronic energy distribution. It is measurable in photoemission experiments [16,54]. In the regular TTM, the electrons follow a Fermi distribution at all times. However, it could be shown that this is not the case after ultrashort laser excitation [13,16,33,35,49,54,55]. Figure 4 shows the non-equilibrium electron distribution at the maximum of the laser pulse calculated by eTTM, comparing the original variant [43] to the model presented in this work (compare Section 2.3). Figure 4a depicts the combined distribution of the thermal background and the non-equilibrium system as described by $f^{\mathrm{tot}}$ in Equation (7), whereas Figure 4b shows only the distribution of the non-equilibrium system Δf. In both curves, a photo-induced step function is observable. The width of the step corresponds to the photon energy $E_{\mathrm{pt}}=1.55\;e\;V$, as expected from the mechanism explained in Section 2. Electrons in occupied states below the Fermi edge absorb photons and are excited to unoccupied states above the Fermi edge. This results in a non-equilibrium distribution, which resembles approximately the curve known from kinetic simulations (compare Section 3.3).

For the joint distribution in Figure 4a, almost no differences between the original eTTM [43] and our implementation are visible. The distributions of the non-equilibrium system in Figure 4b point out the differences between the two implementations in more detail. The original eTTM (red, solid line) has a clearly visible kink at the outer edges of the step. This is an artifact of the energy range of integration being limited to an interval of one photon energy below and above the Fermi edge. Integrating over the whole energy range leads to a more physical smoother curve, as presented in the blue dashed line. The broader integration range leads to a smaller amplitude of the signal as compared to the red solid line, due to the same amount of energy being absorbed for both models. Figure 4b shows stronger changes in the electronic energy distribution in the vicinity of the Fermi edge as compared to the outer edges of the excited region. This is a consequence of the non-zero temperature of the Fermi distribution. At 0 K, the excitation function Equation (3) would show a straight plateau. In addition, at 300 K, the excitation function is more straight, as we will show below in Section 3.3. However, the curve in Figure 4 shows the non-equilibrium electrons at the maximum of the laser pulse, where the equilibrium background has already been considerably heated.

Contrary to the results of the kinetic models, the shape of the DOS does not play a role here. As can be seen in Equation (3), the DOS enters only the amplitude $\delta (t^{\prime })$ but not the shape of the step. This is an oversimplification of the eTTM, which will be discussed in more detail in the following Section 3.3. We have noticed that this neglection of the DOS in the eTTM leads to a deviation from energy and particle conservation laws during irradiation. The deviation is only in the per mille range and reduces for our enlarged integration interval, further supporting the improvements as compared to [43], introduced in Section 2.3.

### 3.3. Comparison to Kinetic Description

As mentioned above, simulations based on the Boltzmann equation [30,31,33,35,48,49] describe the laser-induced non-equilibrium more accurately and thus allow the study of detailed features of the excited electron distribution. However, such kinetic simulations are numerically rather expensive. Here, the eTTM can fill the gap between purely temperature-based models assuming electronic Fermi distributions at all times, and full kinetic simulations of high numerical costs.

To compare the details of the results of such different approaches, we calculated the excited electron distribution with the excitation term of a Boltzmann simulation according to [33], and with the eTTM and TTM, respectively. All calculations start at room temperature ( 300 K) and assume excitation with a δ-like pulse shape of the same wavelength, meaning that s(t=0)≠0ands(t>0)=0 in Equation (4). In this case, the eTTM equations are simplified in a way that the sums in Equations (7) and (9) vanish. The δ-like pulse was chosen to isolate the influence of the absorption from thermalization effects. After the excitation, the electrons in the TTM reach a temperature of 36.418 K. The same energy density has been introduced into the electrons in the Boltzmann kinetic simulation as well as into the non-equilibrium electrons of the eTTM.

**Figure 5:**
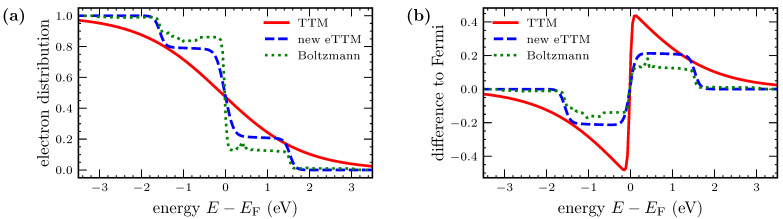
Electron distribution directly after a pulse with a δ-peaked shape. Figure (**a**) shows the distributions with a TTM simulation, an eTTM simulation and a simulation using the Boltzmann collision term for the electron–ion–photon interaction for the same absorbed energy density. The TTM results in a Fermi distribution with elevated temperature. In contrast, the distribution obtained from the eTTM shows two steps, indicating the non-equilibrium. The eTTM and the Boltzmann simulation result in similar distributions. In Figure (**b**), the difference to the initial distribution is shown. The difference between the TTM and the eTTM is striking.

In Figure 5a, the distributions directly after the irradiation are depicted. The TTM approach leads to a thermalized Fermi distribution of elevated temperature. In contrast, both the eTTM and Boltzmann simulations result in a similar step-like non-equilibrium structure. The main step of the Boltzmann-simulated result is less pronounced than that of the eTTM. However, the Boltzmann simulation reveals a second small step, which is due to the two-photon absorption included in this simulation. Furthermore, the Boltzmann-simulated result shows clear features of the DOS imprinted on the distribution [33,56]. In contrast, the eTTM does not include two-photon absorption and is not able to describe the influence of the DOS on the excitation.

Figure 5b shows the difference between the excited electron distributions and the initial Fermi distribution at room temperature. For the eTTM, this difference equals the excitation function Equation (3). Note that the steps of the eTTM and Boltzmann simulations in Figure 5 are more rectangular than those in Figure 4. The initial electron temperature and thus the temperature of the thermalized electrons in the eTTM for the calculations of Figure 5 is 300 K, while for simulations underlying Figure 4, the temperature of the thermal electrons at the maximum of the laser pulse is around 1500 K. This emphasizes the above-explained dependence of the excitation function from the thermal electron temperature and also shows an effect of beginning thermalization during irradiation.

Altogether, we conclude that the absorption behavior can be simulated with the help of the eTTM in a simplified form but in good agreement with the Boltzmann collision term.

### 3.4. Connection to Experiments

For many experiments, the energy-resolved occupation is of interest. With the time-resolved two-photon photoemission, it is possible to observe the dynamics of high-energetic electrons [18,57,58,59]. For instance, photo-electron spectroscopy is a widely used tool to investigate ultrafast processes in microscopic solid state physics [13,22,24,60]. The spectral density is also of importance for element-sensitive studies, e.g., probing magnetic polarization after ultrafast excitation [61,62]. The eTTM can deliver important information for such kinds of experiments via the particle density in a specific energy range in dependence of time.

**Figure 6:**
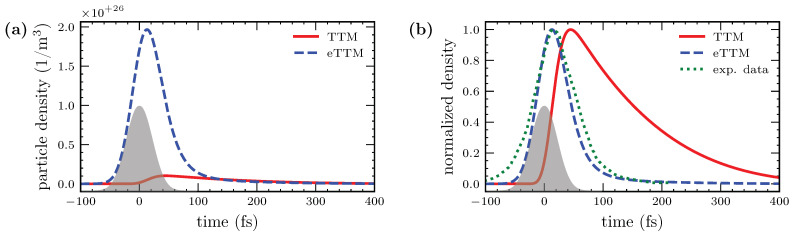
Dynamics of the particle density integrated over the energy range 1 eV to 1.5 eV above the Fermi edge. In Figure (**a**), the result of a TTM simulation is compared to an eTTM simulation. In the eTTM, a significant number of high-energetic electrons are generated directly with the laser pulse and thermalize about 50 fs after the laser. In contrast, the particle density in the TTM only rises with a delay and to a much weaker amplitude. Both spectral densities are normalized in Figure (**b**). Additionally, experimental data from [24] are shown. A good agreement of the curve obtained with the eTTM simulation can be observed, even though the experiment was performed on another material (gold instead of aluminum). In contrast, the spectral density obtained with the TTM shows a much slower decrease.

In Figure 6, the particle number density of electrons in the interval between $E_{1}=1\;e\;V$ and $E_{2}=1.5\;e\;V$ above the Fermi edge is shown. It can be calculated as

(10) $$n_{\mathrm{el}}^{E_{1},E_{2}}=\int_{E_{1}}^{E_{2}}f\left(t,E\right)D\left(E\right)dE$$

with the electron distribution f(t,E) and the DOS D(E). In the case of the eTTM, the total electron distribution given by Equation (7) enters Equation (10); whereas for the case of the TTM, the Fermi distribution of the given temperature $T_{\mathrm{el}}\left(t\right)$ is integrated. The results of the two models, presented in Figure 6a, show a drastic difference. Directly at the beginning of the laser pulse, the number of particles in the given energy window rises sharply when calculated with the eTTM, and falls again rapidly after the end of the laser pulse. In the TTM, the electron density and, thus, the prospected yield of a photoemission experiment increases mainly during the maximum of the laser pulse and decreases on the timescale of electron–phonon coupling. Moreover, the peak of the partial number density in the TTM is about ten-times smaller than in the eTTM. This pronounces the importance of the non-equilibrium distribution in the given energy range.

Figure 6b shows the normalized curves of both cases, pronouncing the different temporal behavior of the spectral densities obtained with the two models. A comparison with experimental data [24] is depicted with a green dotted line. In [24], a time-resolved two-photon photoemission experiment was performed on gold and evaluated in different energy ranges. We extracted the data in Figure 6b from Figure 1 (front pump) of [24] for an energy value of $E-E_{F}=1.3\;e\;V$, which is in the center of the energy interval we integrated for the theoretical curves of Figure 6. The experiment was performed on gold; however, mainly the sp-conduction electrons have been excited. They have a nearly parabolic energy dispersion, as do the aluminum electrons used in our calculations.

The spectral density obtained with the eTTM is in good agreement with the normalized experimental data. In both cases, the laser creates non-equilibrium electrons in the energy range of 1 eV to 1.5 eV above the Fermi edge. Their fast decay has its origin in the thermalization of the non-equilibrium occupation by electron–electron collisions. In contrast, the spectral density of the thermalized system described with the TTM decays much more slowly. The spectral density reaches its maximum with the maximum of the electronic temperature. The decay can be attributed to the slow cooling of the high-temperature electrons by interaction with the phonons. It can be observed also in the results of the eTTM, see Figure 6a. The comparison of the spectral densities in Figure 6 demonstrates the importance of non-equilibrium electron distributions and their strong influence on measurable quantities.

## 4. Summary

We have summarized the extended two-temperature model as introduced by Carpene [44] and modified by Tsibidis [43]. We have shown the excitation and thermalization of the electronic non-equilibrium in comparison to the TTM. Our extension of the energy range of integration in comparison to Tsibidis’ version of this model improves the energy and particle conservation. The eTTM reproduces the typical step-like shape in the distribution function for the laser-excited non-equilibrium distribution. As we have seen from the similarities to the full kinetic description in the absorption behavior, the eTTM has the potential to substitute full Boltzmann calculations for certain research questions. This may be particularly useful in order to obtain an impression of the influence of non-equilibrium effects in dedicated ultrafast experiments. We show this by comparing the eTTM to a time-resolved two-photon photoemission measurement and finding a good agreement between them. Further comparisons, e.g., with the temporal evolution of the results obtained with full Boltzmann collision terms, are ongoing. Already, we can conclude that the extended two-temperature model is a useful tool to describe the electronic non-equilibrium in a numerically favorable way.

## Data Availability

The data that support the findings of this study are available from the corresponding author upon reasonable request.

## Appendix A. Simulation and Material Parameters

In this section the setup of the laser and the parameters for aluminum are listed.

### Appendix A.1. Heat Capacities

The connection between electron distribution $f_{\mathrm{Fermi}}\left(T_{\mathrm{el}},E\right)$ and the internal energy density

(A1) $$u_{\mathrm{el}}=\int_{-\infty }^{\infty }f_{\mathrm{Fermi}}\left(T_{\mathrm{el}},E\right)\;D\left(E\right)\;E\;dE$$

is well-known from solid-state physics text books. From this, the electron heat capacity

(A2) $$c_{\mathrm{el}}=\frac{du}{dT}=\int_{-\infty }^{\infty }\frac{df_{\mathrm{Fermi}}\left(T_{\mathrm{el}},E\right)}{dT}D\left(E\right)EdE$$

is calculated directly from the DOS of electrons, given by D(E) [63,64]. The DOS was calculated with density functional theory and is taken from [65]. The energy zero is set to the Fermi edge; therefore, the integration starts at negative energies.

The phonon heat capacity

(A3) $$c_{\mathrm{ph}}=3n_{\mathrm{ph}}k_{B}=3\frac{\rho_{V}N_{A}}{M}k_{B}=2.5\times 10^{6}J/K/m^{3}$$

is assumed to be constant according to the Dulong–Petit law. Here, the particle density $n_{\mathrm{ph}}$ is calculated with the density $\rho_{V}=2.7\times 10^{6}gm^{-3}$ and the molar mass $M=26.98\;gm^{-3}$ [66].

### Appendix A.2. Laser Setup

A laser pulse with Gaussian profile was used. The intensity

(A4) $$I\left(t\right)=\sqrt{\frac{4\ln(2)}{\pi }}\frac{\Phi }{\tau_{p}}\exp\left[-4\ln\left(2\right){\left(\frac{t}{\tau_{p}}\right)}^{2}\right]$$

at time *t* is given with the fluence $\Phi =15\;Jm^{-2}$ and the full width at half maximum (FWHM), $\tau_{p}=50\;\mathrm{fs}$. With this the laser power density s(t)=(1−R)αI(t) can be calculated. We assume a monochromatic pulse with photon energy $E_{\mathrm{pt}}=1.55\;e\;V$. Here, $\alpha =1.3123\times 10^{8}m^{-1}$ is the absorption coefficient and R=0.8682 the reflectivity of aluminum at room temperature [67]. Note that the optical parameters are actually time-dependent, since they depend on the state of electrons and phonons. For example, they vary with temperature [68,69,70] or band occupation [41,42]. This was, however, not considered in our simulation.

### Appendix A.3. Relaxation Time Parameters

The pre-factor in Equation (6) follows from Fermi liquid theory. It can be calculated as

(A5) $$\tau_{0}=\frac{128}{\sqrt{3}\pi^{2}\omega_{P}}=0.329\mathrm{fs}$$

with the plasma frequency $\omega_{P}=14.98\;e\;V/\hbar$ [44,50,71]. Note that this pre-factor of Equation (6) results in thermalization times up to picosecond timescale at the Fermi edge.

The electron–phonon relaxation time appearing in Equation (5) results as $\tau_{\mathrm{el}*-\mathrm{ph}}=299.22\mathrm{fs}$, according to [19,44] with the data from [72,73].

### Appendix A.4. Miscellaneous

We took the electron–phonon coupling parameter to be constant and used a value of $g=2.6148\times 10^{17}JK^{-1}s^{-1}m^{-3}$, taken from [65] at 1000 K. Note that a constant coupling parameter is an approximation. Generally, the strength of electron–phonon coupling varies with temperature [46,52,65].

An FCC crystal with lattice constant $4.05\times 10^{-10}$ m was considered [65].
